# Metabolic Pathways in Hydrocephalus: Profiling with Proteomics and Advanced Imaging

**DOI:** 10.3390/metabo14080412

**Published:** 2024-07-27

**Authors:** Laura May Davis, Misun Hwang

**Affiliations:** 1 Clinical Research Core, Children’s Hospital of Philadelphia, Philadelphia, PA 19104, USA; davisl19@chop.edu; 2 Department of Radiology, Children’s Hospital of Philadelphia, University of Pennsylvania, Philadelphia, PA 19104, USA

**Keywords:** hydrocephalus, contrast-enhanced ultrasound, proteomics, super-resolution imaging

## Abstract

Hemorrhagic hydrocephalus is a common pathology in neonates with high mortality and morbidity. Current imaging approaches fail to capture the mechanisms behind its pathogenesis. Here, we discuss the processes underlying this pathology, the metabolic dysfunction that occurs as a result, and the ways in which these metabolic changes inform novel methods of clinical imaging. The imaging advances described allow earlier detection of the cellular and metabolic changes, leading to better outcomes for affected neonates.

## 1. Introduction

Hydrocephalus—the build-up of cerebrospinal fluid (CSF) produced by the choroid plexus cells lining the ventricular system—contributes a global disability weight greater than that of tuberculosis, rheumatic heart disease, and blindness combined. It has a higher incidence in low- and middle-income countries (123 per 100,000 births vs. 79 per 100,000 births in high-income countries) [1,2]. The prolonged increases in intracranial pressure (ICP) that can result from hydrocephalus are correlated with negative short- and long-term neuro-behavioral outcomes, making early detection and intervention imperative [3].

Understanding of the pathophysiological processes of hydrocephalus has developed from an initial understanding of it as a disease of reduced CSF drainage to a more nuanced view [4]. In this model, acute hydrocephalus is caused by sudden-onset blockage of CSF outflow (e.g., a large clot in the ventricular system), whilst chronic hydrocephalus occurs as a result of loss of intracranial compliance and mismatch between the CSF and brain parenchymal pressure [5,6]. More recently, research has focused on the role of excessive CSF production, and the role inflammatory pathway activation has in this [4,5,6,7]. Approaches to the understanding of these processes have progressed from the animal models of Dandy, in which plexotomy allowed the role of the choroid plexus to be elucidated, to the direct measurement of CSF and brain parenchymal pressure, and more recently, proteomic approaches have been taken to allow a better understanding of the cellular and metabolic processes involved [3,8,9,10,11].

Whist histopathological change is seen in neonatal brains with hemorrhagic hydrocephalus and includes loss of subventricular stem cells and ependymal cells, as well as astrocyte invasion into areas of hemorrhage, these changes are not acute; they develop over days to weeks. As a result, at autopsy, humans and animal models with acute hydrocephalus can show minimal histological change despite significant metabolic disturbance [2,12,13,14,15]. However, prior to histological change being appreciable, there are changes in intracranial compliance, loss of normal CSF flow, and activation of inflammatory pathways and a period of cellular dysfunction [12]. If these changes can be detected early, prior to the onset of histological change, through a blood, CSF, or imaging biomarker, intervention can occur in the window prior to histological change, potentially resulting in better short- and long-term neurodevelopmental outcomes. This review will give an overview of prior and current research into neonatal hydrocephalus and the metabolic derangements and sequalae that occur because of it. We will then consider novel imaging approaches and their associated diagnostic and therapeutic potential.

## 2. Causes of Neonatal Hydrocephalus

In the premature neonate, hemorrhage is the most common cause of hydrocephalus due to the immaturity of the choroid plexus vasculature, which is predisposed to bleeding [16,17]. Prior to 34 weeks, vessels in the germinal matrix do not have adequate surrounding stroma and have a propensity to rupture and bleed [2,17]. Extremely preterm infants require extensive hemodynamic support and are exposed to periods of cardiovascular instability, meaning that the vessels undergo pressure changes which can precipitate hemorrhage [2]. In total, 15 to 35% of preterm babies who have intraventricular hemorrhage subsequently develop hydrocephalus [18].

Other causes include infection (e.g., bacterial meningitis, tuberculosis) and tumors causing CSF outflow obstruction [2], as well as failure to receive antenatal steroid treatment [19]. Syndromic causes include X-linked recessive hydrocephalus, a group of related pathologies arising from mutations in the L1 cell adhesion molecule protein (a neuronal cellular adhesion molecule), as well as myelomeningocele and other neural tube defects [2]. Hydranencephaly, developing from an in utero insult to the developing brain that results in infarction of the brain tissue, is an additional cause of congenital hydrocephalus [2]. The remainder of this review will focus on hemorrhagic hydrocephalus as the most common cause in the neonate.

## 3. Causes of Metabolic Dysfunction in Hemorrhagic Hydrocephalus

**Figure 1:**
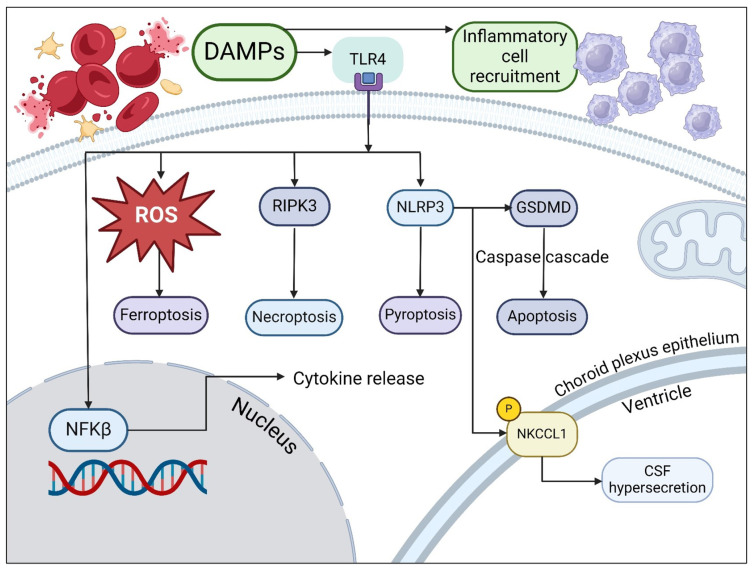
Summary of pathways involved in hemorrhagic hydrocephalus. Damage-associated molecular patterns (DAMPs) activating toll-like receptors (TLRs), as well as leading to inflammatory cell recruitment, causing (1) reactive oxygen species generation; (2) receptor-activating protein kinase 3 (RIPK3) activation with resultant necroptosis; (3) NOD-like receptor family pyrin domain-containing 3 (NLRP3) inflammasome activation and resultant pyroptosis, Gasdermin D activation leading to apoptosis, and phosphorylation of sodium, potassium chloride co-transporter-1 (NKCCL1) by the NLRP3 inflammasome complex in the choroid plexus epithelium, resulting in CSF hypersecretion (GSDMD: Gasdermin D; NFKβ: nuclear factor kappa beta; ROS: reactive oxygen species (created with BioRender.com, accessed on 12 June 2024)).

The pathways that are involved in post-hemorrhagic hydrocephalus are summarized in Figure 1. Following hemorrhage, red blood cells are present in supra-physiological numbers in the CSF and undergo lysis, leading to the presence of damage-associated molecular patterns (DAMPs) [20]. DAMPs include heat shock proteins, blood breakdown proteins, (peroxiredoxin, hemoglobin and its degradation products, including iron and carbonic anhydrase) and high-mobility group box 1 protein (HMGB1), a pro-inflammatory molecule [21,22,23,24]. Hemoglobin products complex with haptoglobin and are endocytosed by cells of the reticuloendothelial system via the receptor CD163, a monocyte and macrophage receptor upregulated by inflammation, leading to inflammatory cell recruitment [25]. Iron products cause the formation of reactive oxygen species, which accumulate within cells, leading to oxidative injury, swelling and ferroptosis—iron-dependent cell death [26,27]. Release of HMGB1 occurs following tissue injury, when it is upregulated in damaged cells and actively secreted by damaged neurons and microglia [28,29].

Following interaction with DAMPs and Toll-like receptors, in particular TLR4, which is predominantly expressed in the brain, intracellular signaling occurs in microglia that leads to activation of NFKβ [21]. TLR4 interacts with myeloid differentiation primary response gene 88 [MyD88], leading to the recruitment of death-domain-containing molecules and subsequent cytokine release, activation of NFKβ due to TNF-associated factor 6 (TRAF6) activation, and necroptosis [30]. TLR4 activation also leads to induction of interferon regulatory factor transcription 3, resulting in interferon release and NFKβ activation [30,31]. TLR4 activation also leads to necroptotic cell death via activation of RIPK3, a form of programmed cell death that results in pro-inflammatory mediator and DAMP release, causing further inflammatory activation [32,33].

TLR interaction with DAMPs and subsequent interaction with MyD88 and reactive oxygen species lead to activation of the nucleotide oligomerization domain-like receptor family pyrin domain-containing 3 (NLRP3) inflammasome, a complex upregulated in astrocytes and microglia following inflammatory activation [34,35,36]. Following activation of the NLRP3 inflammasome, cleavage of Gasdermin D (GSDMD) occurs, resulting in cell membrane pore formation and cell death via pyroptosis [37]. NLRP3-driven cytokine release leads to disruption of the blood–CSF barrier, which can be partly ameliorated by glucocorticoid immunosuppression; in a post-hemorrhagic mouse model, expression of tight junction proteins was preserved in mice given immunosuppression relative to those without [38]. NLRP3 was also shown to increase CSF secretion in a post-hemorrhagic hydrocephalic rat model. CSF is primarily produced by epithelial cells of the choroid plexus, a row of tightly organized cuboidal cells bound together by tight junctions surrounding clusters of fenestrated capillaries [12,39]. One transporter responsible for the close control of CSF volume and electrolyte balance is the sodium, potassium, and chloride co-transporter, NKCC1, which sits at the luminal membrane of the blood–CSF barrier and contributes to around 50% of CSF production by transporting Na^+^, Cl^−^ and K^+^ across an osmotic gradient [34]. Proteomic analysis of post-hemorrhagic rats demonstrates increased NLRP expression, whilst in an NLRP3 knockout model, there was a decrease in inflammation, CSF secretion, and hydrocephalus [34]. NLRP3-knockout rats showed reduced NKCC1 phosphorylation; NLRP3 is thought to result in phosphorylation of NKCC1 and subsequent caspase-1 activation [34]. Administration of a caspase-1 inhibitor (VX765) intranasally in mice resulted in attenuated neurological deficits, less edema on imaging, lower rates of intracranial hypertension at 24 and 72 h, and fewer macroscopic blood clots on the brain surface. CSF flow analysis in rats using injected dye showed that with caspase-1 inhibition, there was marked improvement in CSF movement in the acute phase [40]. The caspase activation that occurs as a result of NLRP3 activation also results in cell death via apoptosis, a form of predominantly non-inflammatory programmed cell death characterized by fragmentation of the nucleus, bleb formation in the plasma membrane, and ultimately apoptotic cell death [37]. Immunofluorescence staining demonstrates the presence of cleaved caspase-1 in microglia, astrocytes, and endothelial cells near the sites of artery rupture, indicative of apoptotic pathway activation [40].

## 4. Neurometabolic Dysfunction

Just as the inflammatory activation described above affects both parenchyma and CSF, there is also evidence of neurometabolic dysfunction in both CSF and brain parenchyma. As a result of NFKβ transcription, its subunit, p-65, was shown in a rat model to be upregulated in choroid plexus cells. An increase in uptake of immunoglobulin G by barrier cells was also demonstrated, in keeping with altered barrier permeability [41]. Parallel changes have been demonstrated in CSF microdialysate, which is more accessible and less invasive to obtain than neural tissue. CSF biomarkers of metabolic derangement include the lactate-to-pyruvate ratio, which has been shown in a pig model to increase with rises in ICP, decreasing cerebral perfusion pressure and reducing brain tissue oxygenation [42,43]. The alteration in the lactate-to-pyruvate ratio signals dysregulation in cellular metabolic pathways and a shift towards anaerobic metabolism [44]. Glucose levels are also altered in raised ICP, with cortical glucose markedly decreasing when ICP increases and cerebral perfusion decreases, as a result of these substrates being utilized in anaerobic metabolism [43,45]. Glutamate and glycerol rise in parallel with increasing ICP [43]. The rise in glycerol precedes the increase in glutamate, suggesting that initial rises are due to glycolysis, with further increases resulting from membrane breakdown and glycerophospholipid release [46]. The rise in glutamate correlates with a discrete gradient increase in the lactate-to-pyruvate ratio, indicating failure of energy-dependent ion pumps crucial to neuronal function and marking the occurrence of irreversible neurometabolic dysfunction and neuronal damage [47,48]. One difference to be aware of in the fetal and neonatal population is the fact that in the fetal and neonatal brain, there is a shift—beginning in the third trimester—from anaerobic glycolysis to aerobic metabolism in the fetal and neonatal brain, so the above findings cannot be directly applied to the neonatal population [49].

## 5. Imaging of Neurometabolic Derangement and Future Directions

Despite the multitude of pathological pathways and potential biomarkers in hydrocephalus, there is no widely used imaging technique that informs the clinician about the pathophysiology or the metabolic impact of this disease [2,50,51]. Conventional imaging is performed with ultrasound and fetal and neonatal magnetic resonance imaging (MRI). Standard evaluation of neonatal hydrocephalus with transcranial ultrasound uses serial measurements of ventricular size to inform the need for intervention. Ventricular size, however, is at best a proxy for intracranial pressure elevation, and the presence of ventriculomegaly on fetal imaging does not always correlate with post-natal hydrocephalus. In these cases, serial ultrasound measurements are performed in the immediate post-natal period to understand whether intervention is required. These measurements fail to deliver information regarding the actual intracranial pressure, or any impact on the neural tissue and the biochemical pathways [52]. Frequently, by the time there is significant ventriculomegaly, irreversible damage has already occurred to the brain tissue. In other cases, intracranial pressure is elevated without appreciable ventricular enlargement [53]. While intracranial pressure can be closely monitored with invasive sensors, these are associated with significant mortality and morbidity [54].

Imaging methods for ascertaining neurometabolic status exist in the research setting and include oxygen extraction fraction mapping using MRI [55]. This approach was studied in adult humans with normal-pressure hydrocephalus, showing that the oxygen extraction fraction was significantly lower than healthy controls, and this was applicable to both the whole brain and distinct regions (gray matter, thalamus, caudate and putamen) [55]. This presents an alternative to the gold standard method of brain metabolism assessment: positron emission tomography using oxygen-15-labeled radiopharmaceuticals [56]. Another approach is the use of near-infrared spectroscopy (NIRS), in which the light waves are absorbed by oxy- and de-oxyhemoglobin at different wavelengths, and a derivation of their respective concentrations can be made by measuring the light intensities returned to the transmitter [56]. Given the thinness of the neonatal skull, NIRS has applicability as a bedside test and is affordable, but it does not allow absolute cerebral metabolic rate of oxygenation, unlike other methods [56].

Other approaches to imaging this population have focused on non-invasive means of monitoring intracranial pressure, given that there is a clear linkage between the degree of raised intracranial pressure and neurometabolic dysfunction. These approaches include transcranial Doppler, contrast-enhanced ultrasound techniques, and MRI techniques, among others. The role of MRI in the assessment of intracranial pressure has been investigated in baboon and human subjects. MRI-derived measurements of CSF and blood flow were used to ascertain the volume change in CSF (a derivation based on the Monro–Kellie doctrine that the intracranial contents are a fixed volume) and the change in pressure (derived from CSF velocity); these were used to calculate an elastance index (a ratio of pressure to volume change). This was shown to have good correlation with invasively measured pressures when performed serially in both healthy and raised-ICP subjects, with normal MRI-derived ICP correlating with symptomatic resolution [57]. However, at higher levels of raised ICP, there was less correlation between the actual ICP and that measured by the MRI, making this less useful as an initial diagnostic tool [58].

**Figure 2:**
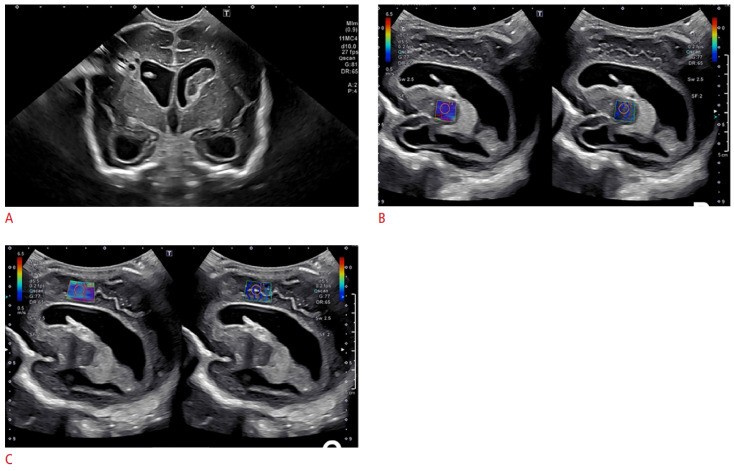
Ultrasound elastography of a 2-month-old, former 28-week and 4-day-old male infant following shunt placement for treatment of post-hemorrhagic hydrocephalus. (**A**) Grayscale coronal ultrasound image showing a catheter placed in the right lateral ventricle. Cystic change is present in the right periventricular white matter in keeping with prior infarct. Following shunt placement, elastography was monitored and showed (**B**) the right basal ganglia, with a value of 1.55 m/s and (**C**) the periventricular white matter with a value of 2.18 m/s. (Images under creative commons license, https://creativecommons.org/licenses/by-nc/4.0/, accessed on 25 July 2024) [68].

Shear-wave elastography using acoustic radiation force impulse imaging (ARFI)—where a high-intensity sonic wave is passed through tissue to ascertain tissue stiffness via waves at right angles to the initial force—has been used to assess neonatal brains [59]. Unlike MRI, it has the advantage of being portable and able to be performed at the bedside of critically ill neonates [60]. Its utility has been demonstrated in both stroke and traumatic brain injury [61,62]. Studies have used ARFI to show that in normal neonates, elastic values increase with age as a result of increasing myelination [63,64]. In a comparison of normal and hydrocephalic infants, increased stiffness was seen in the hydrocephalic group, suggesting that additional changes in the tissue are being detected and making ARFI a useful adjunct to bedside imaging in these patients (Figure 2) [65]. This technique has the potential to be applied in a more widespread clinical context. In addition, there has been early research into the use of elastography in the fetal brain in animal models, suggesting that future use could extend to the fetus with hydrocephalus, allowing clinicians to understand the impact on the brain before the child is delivered [66,67].

**Figure 3:**
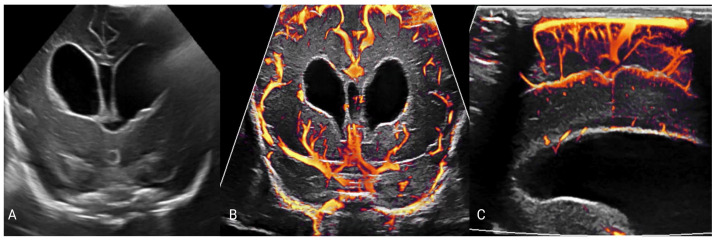
Series of images in a 22-day-old preterm female with hydrocephalus secondary to intraventricular hemorrhage. (**A**) shows the coronal view in grayscale at the level of the lateral ventricles, showing a marked increase in ventricular size and layering blood products within the ventricles. (**B**,**C**) show microvascular imaging of the slow-flowing vessels in coronal (**B**) and sagittal (**C**) planes, with relative velocity shown by faster flow in yellow and less fast in orange to red (images under creative commons license https://creativecommons.org/licenses/by-nc/4.0/ accessed on 25 July 2024 [68]).

Doppler techniques looking at the hemodynamics of the intracranial vessels show a correlation between asymmetrical dilatation of the lateral ventricles and the basal resistive index in the anterior cerebral artery, with decreasing ventricular size after intervention correlating with a decrease in the resistive index in this vessel [69]. However, Doppler parameters in these macrovessels are subject to extracranial factors, making this a suboptimal means of assessing ICP [70]. Imaging of the microvessels using advanced Doppler techniques uses filters to visualize only the slow flow in the smaller intracranial vessels (Figure 3), which show a marked reduction in flow with raised intracranial pressure that is disproportionate to that seen in macrovessels [71,72]. Advanced Doppler techniques can also be used to demonstrate altered CSF in post-hemorrhagic hydrocephalus [68].

**Figure 4:**
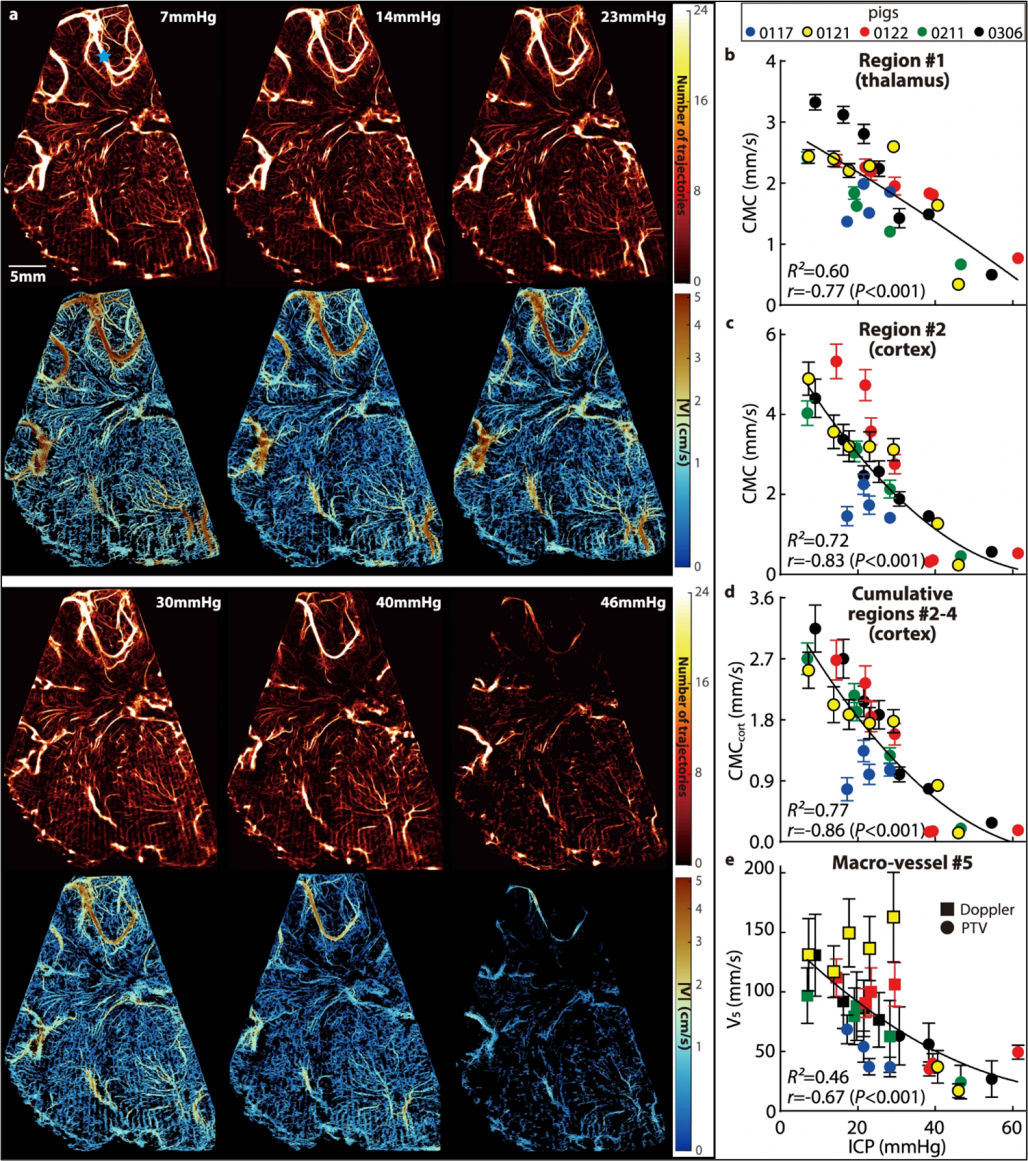
(**a**) Cerebral blood vessels (**top** row) and the corresponding velocity distributions (**bottom** row) for piglet 0121, demonstrating the effect of increasing intracranial pressure on the cerebral perfusion as detected by super-resolution ultrasound. (**b**–**d**): Relationships between intracranial pressure and the cerebral microcirculation (CMC, original cohort) parameters in the thalamus (**b**), in cortical subregion 2 (**c**), and in all three cortical subregions combined ((**d**), CMCcort). (**e**) Relationships between the intracranial pressure and the time-averaged velocity (V5) in the macro blood vessel are marked with a blue star in (**a**). For (**b**–**e**), data are presented as mean +/− SD, where the error bars show the temporal standard deviations of CMCs or V5 over 20 cardiac phases; several standard deviation values for high ICP are too small to be shown. The curves are least-square fitted quadratic functions, with the corresponding coefficient of determination (R2) shown on each plot. Also presented are the correlation coefficients (r) and the corresponding *p* values between the CMCs or V5 and the ICP. Images under creative commons license, http://creativecommons.org/licenses/by/4.0/ (accessed on 24 May 2024) [78].

Contrast-enhanced ultrasound allows evaluation of both microvasculature and the effects of raised intracranial pressure on the brain parenchyma. Contrast-enhanced ultrasound uses intravenous microbubbles—2–3 micrometers, slightly smaller than a red blood cell—to allow radiation-free, accessible, and dynamic evaluation of the neonatal brain. Tracking these highly echogenic bubbles with super-resolution ultrasound allows the creation of a map of the cerebral circulation to a far higher resolution than would be achievable with conventional ultrasound, MRI, or CT [73,74,75]. The bubbles also allow measurements of blood flow velocity within microvessels that are not evaluable with usual Doppler flow measurement techniques [76,77]. In a porcine hydrocephalus model, this has been used to perform intracranial circulatory mapping across a range of raised ICPs, demonstrating regional alterations in perfusion as ICP increases (Figure 4) [78]. From these data, an index of regional microvascular perfusion can be derived that has a significant functional relationship with ICP, and has potential as an imaging biomarker of raised intracranial pressure [78]. In addition, reduced cerebral microcirculation in this model was correlated with ischemia (indicated by an increased lactate-to-pyruvate ratio on CSF microdialysis), suggesting that this imaging approach allows the detection of metabolic derangement as well as structural change [78]. This combination of bubble tracking and velocity is a promising means of assessing the hydrocephalic neonate, and further work to evaluate the histopathological and proteomic correlates of these imaging features will allow a better understanding of the changes occurring on a cellular level that correspond with these imaging features. Additional promising future applications are the use of theragnostic microbubbles, allowing both diagnosis of pathology and its simultaneous treatment through drugs encapsulated within the microbubble that can be locally released with a sound wave that causes bubble disruption [79].

## 6. Conclusions

Hydrocephalus in the neonate has multiple triggers and complex pathophysiological pathways that converge on widespread neurometabolic dysfunction and, at a certain point, irreversible brain damage. Proteomic analysis allows the elucidation of these pathways and their implications for neurometabolism, as well as the development of imaging techniques that demonstrate both the neurometabolic dysfunction and its pathological sequelae. Future work will focus on correlating proteomic expression with imaging findings to allow earlier, non-invasive detection of the cellular changes wrought by hydrocephalus.

## Data Availability

No new data were created or analyzed in this study. Data sharing is not applicable to this article.

## References

[1] Dewan M.C., Rattani A., Mekary R., Glancz L.J., Yunusa I., Baticulon R.E., Fieggen G., Wellons J.C., Park K.B., Warf B.C. (2018). Global hydrocephalus epidemiology and incidence: Systematic review and meta-analysis. J. Neurosurg..

[2] Flanders T.M., Billinghurst L., Flibotte J., Heuer G.G. (2018). Neonatal Hydrocephalus. NeoReviews.

[3] Rostgaard N., Olsen M.H., Lolansen S.D., Norager N.H., Plomgaard P., MacAulay N., Juhler M. (2023). Ventricular CSF proteomic profiles and predictors of surgical treatment outcome in chronic hydrocephalus. Acta Neurochir..

[4] Dandy W.E. (1919). Experimental hydrocephalus. Ann. Surg..

[5] Greitz D. (2007). Paradigm shift in hydrocephalus research in legacy of Dandy’s pioneering work: Rationale for third ventriculostomy in communicating hydrocephalus. Child’s Nerv. Syst..

[6] Bering E.A. (1962). Circulation of the cerebrospinal fluid. Demonstration of the choroid plexuses as the generator of the force for flow of fluid and ventricular enlargement. J. Neurosurg..

[7] Karimy J.K., Duran D., Hu J.K., Gavankar C., Gaillard J.R., Bayri Y., Rice H., DiLuna M.L., Gerzanich V., Marc Simard J. (2016). Cerebrospinal fluid hypersecretion in pediatric hydrocephalus. Neurosurg. Focus.

[8] Chen S., Lin D., Liu P., Liu Q., Li M., Han W., Wang X., Zhang W., Song H., Li Z. (2022). Quantitative assessment of renal perfusion in children with UPJO by contrast enhanced ultrasound: A pilot study. J. Pediatr. Urol..

[9] Yuan L., Zou D., Yang X., Chen X., Lu Y., Zhang A., Zhang P., Wei F. (2021). Proteomics and functional study reveal kallikrein-6 enhances communicating hydrocephalus. Clin. Proteom..

[10] Waybright T., Avellino A.M., Ellenbogen R.G., Hollinger B.J., Veenstra T.D., Morrison R.S. (2010). Characterization of the human ventricular cerebrospinal fluid proteome obtained from hydrocephalic patients. J. Proteom..

[11] Yu G., Zhang Y., Ning B. (2021). Reactive astrocytes in central nervous system injury: Subgroup and potential therapy. Front. Cell. Neurosci..

[12] Karimy J.K., Reeves B.C., Damisah E., Duy P.Q., Antwi P., David W., Wang K., Schiff S.J., Limbrick D.D., Alper S.L. (2020). Inflammation in acquired hydrocephalus: Pathogenic mechanisms and therapeutic targets. Nat. Rev. Neurol..

[13] Cherian S., Whitelaw A., Thoresen M., Love S. (2004). The pathogenesis of neonatal post-hemorrhagic hydrocephalus. Brain Pathol..

[14] Hirayama A. (1980). Histopathological study of congenital and acquired experimental hydrocephalus. Brain Dev..

[15] Holste K.G., Xia F., Ye F., Keep R.F., Xi G. (2022). Mechanisms of neuroinflammation in hydrocephalus after intraventricular hemorrhage: A review. Fluids Barriers CNS.

[16] Ballabh P., Xu H., Hu F., Braun A., Smith K., Rivera A., Lou N., Ungvari Z., Goldman S.A., Csiszar A. (2007). Angiogenic inhibition reduces germinal matrix hemorrhage. Nat. Med..

[17] Gould S.J., Howard S. (1988). Glial differentiation in the germinal layer of fetal and preterm infant brain: An immunocytochemical study. Pediatr. Pathol./Affil. Int. Paediatr. Pathol. Assoc..

[18] Allard J.B., Duan C. (2018). IGF-Binding Proteins: Why Do They Exist and Why Are There So Many?. Front. Endocrinol..

[19] Linder N., Haskin O., Levit O., Klinger G., Prince T., Naor N., Turner P., Karmazyn B., Sirota L. (2003). Risk factors for intraventricular hemorrhage in very low birth weight premature infants: A retrospective case-control study. Pediatrics.

[20] Miyake K. (2007). Innate immune sensing of pathogens and danger signals by cell surface Toll-like receptors. Semin. Immunol..

[21] Kwon M.S., Woo S.K., Kurland D.B., Yoon S.H., Palmer A.F., Banerjee U., Iqbal S., Ivanova S., Gerzanich V., Simard J.M. (2015). Methemoglobin is an endogenous toll-like receptor 4 ligand-relevance to subarachnoid hemorrhage. Int. J. Mol. Sci..

[22] Chen T., Tan X., Xia F., Hua Y., Keep R.F., Xi G. (2021). Hydrocephalus Induced by Intraventricular Peroxiredoxin-2: The Role of Macrophages in the Choroid Plexus. Biomolecules.

[23] Huang S., Hu W., Rao D., Wu X., Bai Q., Wang J., Chu Z., Xu Y. (2022). RIPK3-Dependent Necroptosis Activates MCP-1-Mediated Inflammation in Mice after Intracerebral Hemorrhage. J. Stroke Cerebrovasc. Dis..

[24] Guo F., Hua Y., Wang J., Keep R.F., Xi G. (2012). Inhibition of carbonic anhydrase reduces brain injury after intracerebral hemorrhage. Transl. Stroke Res..

[25] Ascenzi P., Bocedi A., Visca P., Altruda F., Tolosano E., Beringhelli T., Fasano M. (2005). Hemoglobin and heme scavenging. IUBMB Life.

[26] Cheng T., Wang C., Lu Q., Cao Y., Yu W., Li W., Liu B., Gao X., Lü J., Pan X. (2022). Metformin inhibits the tumor-promoting effect of low-dose resveratrol, and enhances the anti-tumor activity of high-dose resveratrol by increasing its reducibility in triple negative breast cancer. Free Radic. Biol. Med..

[27] Dixon S.J., Lemberg K.M., Lamprecht M.R., Skouta R., Zaitsev E.M., Gleason C.E., Patel D.N., Bauer A.J., Cantley A.M., Yang W.S. (2012). Ferroptosis: An iron-dependent form of nonapoptotic cell death. Cell.

[28] Kim J.B., Sig Choi J., Yu Y.M., Nam K., Piao C.S., Kim S.W., Lee M.H., Han P.L., Park J.S., Lee J.K. (2006). HMGB1, a novel cytokine-like mediator linking acute neuronal death and delayed neuroinflammation in the postischemic brain. J. Neurosci..

[29] Paudel Y.N., Angelopoulou E., Piperi C., Othman I., Shaikh M.F. (2020). HMGB1-Mediated Neuroinflammatory Responses in Brain Injuries: Potential Mechanisms and Therapeutic Opportunities. Int. J. Mol. Sci..

[30] Lu Y.C., Yeh W.C., Ohashi P.S. (2008). LPS/TLR4 signal transduction pathway. Cytokine.

[31] Honda K., Taniguchi T. (2006). IRFs: Master regulators of signalling by Toll-like receptors and cytosolic pattern-recognition receptors. Nat. Rev. Immunol..

[32] Cho Y.S., Challa S., Moquin D., Genga R., Ray T.D., Guildford M., Chan F.K.M. (2009). Phosphorylation-driven assembly of the RIP1-RIP3 complex regulates programmed necrosis and virus-induced inflammation. Cell.

[33] Liu C., Chen Y., Cui W., Cao Y., Zhao L., Wang H., Liu X., Fan S., Huang K., Tong A. (2021). Inhibition of neuronal necroptosis mediated by RIP1/RIP3/MLKL provides neuroprotective effects on kaolin-induced hydrocephalus in mice. Cell Prolif..

[34] Zhang Z., Tan Q., Guo P., Huang S., Jia Z., Liu X., Feng H., Chen Y. (2022). NLRP3 inflammasome-mediated choroid plexus hypersecretion contributes to hydrocephalus after intraventricular hemorrhage via phosphorylated NKCC1 channels. J. Neuroinflamm..

[35] Walsh J.G., Muruve D.A., Power C. (2014). Inflammasomes in the CNS. Nat. Rev. Neurosci..

[36] Groslambert M., Py B.F. (2018). Spotlight on the NLRP3 inflammasome pathway. J. Inflamm. Res..

[37] Huang Y., Xu W., Zhou R. (2021). NLRP3 inflammasome activation and cell death. Cell. Mol. Immunol..

[38] Cheng S., Gao W., Xu X., Fan H., Wu Y., Li F., Zhang J., Zhu X., Zhang Y. (2016). Methylprednisolone sodium succinate reduces BBB disruption and inflammation in a model mouse of intracranial haemorrhage. Brain Res. Bull..

[39] Robert S.M., Reeves B.C., Kiziltug E., Duy P.Q., Karimy J.K., Mansuri M.S., Marlier A., Allington G., Greenberg A.B.W., DeSpenza T. (2023). The choroid plexus links innate immunity to CSF dysregulation in hydrocephalus. Cell.

[40] Fang Y., Wang X., Lu J., Shi H., Huang L., Shao A., Zhang A., Liu Y., Ren R., Lenahan C. (2022). Inhibition of caspase-1-mediated inflammasome activation reduced blood coagulation in cerebrospinal fluid after subarachnoid haemorrhage. EBioMedicine.

[41] Simard P.F., Tosun C., Melnichenko L., Ivanova S., Gerzanich V., Simard J.M. (2011). Inflammation of the choroid plexus and ependymal layer of the ventricle following intraventricular hemorrhage. Transl. Stroke Res..

[42] Purins K., Enblad P., Wiklund L., Lewén A. (2012). Brain tissue oxygenation and cerebral perfusion pressure thresholds of ischemia in a standardized pig brain death model. Neurocritical Care.

[43] Zoremba N., Schnoor J., Berens M., Kuhlen R., Rossaint R. (2007). Brain metabolism during a decrease in cerebral perfusion pressure caused by an elevated intracranial pressure in the porcine neocortex. Anesth. Analg..

[44] Magnoni S., Ghisoni L., Locatelli M., Caimi M., Colombo A., Valeriani V., Stocchetti N. (2003). Lack of improvement in cerebral metabolism after hyperoxia in severe head injury: A microdialysis study. J. Neurosurg..

[45] Ljunggren B., Schutz H., Siesjö B.K. (1974). Changes in energy state and acid-base parameters of the rat brain during complete compression ischemia. Brain Res..

[46] Marklund N., Salci K., Lewén A., Hillered L. (1997). Glycerol as a marker for post-traumatic membrane phospholipid degradation in rat brain. NeuroReport.

[47] Choi D.W., Hartley D.M. (1993). Calcium and glutamate-induced cortical neuronal death. Res. Publ.-Assoc. Res. Nerv. Ment. Dis..

[48] Choi D.W. (1988). Glutamate neurotoxicity and diseases of the nervous system. Neuron.

[49] du Plessis A.J. (2009). Cerebral blood flow and metabolism in the developing fetus. Clin. Perinatol..

[50] Foss-Skiftesvik J., Andresen M., Juhler M. (2013). Childhood hydrocephalus - is radiological morphology associated with etiology. SpringerPlus.

[51] Horst K.K., Leschied J.R., Janitz E.M., Kim J.S., Narayanan S., Setty B.N., Birkemeier K., Sintim-Damoa A., Lampl B.S., Pomeranz C.B. (2023). Neonatal neurosonography practices: A survey of active Society for Pediatric Radiology members. Pediatr. Radiol..

[52] Behrendt N., Zaretsky M.V., West N.A., Galan H.L., Crombleholme T.M., Meyers M.L. (2017). Ultrasound versus MRI: Is there a difference in measurements of the fetal lateral ventricles?. J. Matern.-Fetal Neonatal Med..

[53] Zhang Z., Hwang M., Kilbaugh T.J., Sridharan A., Katz J. (2022). Cerebral microcirculation mapped by echo particle tracking velocimetry quantifies the intracranial pressure and detects ischemia. Nat. Commun..

[54] Zhang X., Medow J.E., Iskandar B.J., Wang F., Shokoueinejad M., Koueik J., Webster J.G. (2017). Invasive and noninvasive means of measuring intracranial pressure: A review. Physiol. Meas..

[55] Zhuang H., Cho J., Chiang G.C.Y., Kovanlikaya I., Heier L.A., Dyke J.P., Wang Y. (2023). Cerebral oxygen extraction fraction declines with ventricular enlargement in patients with normal pressure hydrocephalus. Clin. Imaging.

[56] Liu P., Chalak L.F., Lu H. (2014). Non-invasive assessment of neonatal brain oxygen metabolism: A review of newly available techniques. Early Hum. Dev..

[57] Alperin N.J., Lee S.H., Loth F., Raksin P.B., Lichtor T. (2000). MR-Intracranial pressure (ICP): A method to measure intracranial elastance and pressure noninvasively by means of MR imaging: Baboon and human study. Radiology.

[58] Glick R.P., Niebruegge J., Lee S.H., Egibor O., Lichtor T., Alperin N. (2006). Early experience from the application of a noninvasive magnetic resonance imaging-based measurement of intracranial pressure in hydrocephalus. Neurosurgery.

[59] Ozturk A., Grajo J.R., Dhyani M., Anthony B.W., Samir A.E. (2018). Principles of ultrasound elastography. Abdom. Radiol..

[60] Bailey C., Huisman T.A.G.M., de Jong R.M., Hwang M. (2017). Contrast-Enhanced Ultrasound and Elastography Imaging of the Neonatal Brain: A Review. J. Neuroimaging.

[61] Xu Z.S., Yao A., Chu S.S., Paun M.K., McClintic A.M., Murphy S.P., Mourad P.D. (2014). Detection of mild traumatic brain injury in rodent models using shear wave elastography: Preliminary studies. J. Ultrasound Med..

[62] Martin A., Schneiderman J., Helenowski I.B., Morgan E., Dilley K., Danner-Koptik K., Hatahet M., Shimada H., Cohn S.L., Kletzel M. (2014). Secondary malignant neoplasms after high-dose chemotherapy and autologous stem cell rescue for high-risk neuroblastoma. Pediatr. Blood Cancer.

[63] Su Y., Ma J., Du L., Xia J., Wu Y., Jia X., Cai Y., Li Y., Zhao J., Liu Q. (2015). Application of acoustic radiation force impulse imaging (ARFI) in quantitative evaluation of neonatal brain development. Clin. Exp. Obstet. Gynecol..

[64] El-Ali A.M., Subramanian S., Krofchik L.M., Kephart M.C., Squires J.H. (2020). Feasibility and reproducibility of shear wave elastography in pediatric cranial ultrasound. Pediatr. Radiol..

[65] deCampo D., Hwang M. (2018). Characterizing the neonatal brain with ultrasound elastography. Pediatr. Neurol..

[66] Pavan L., Gasser B., Maronezi M.C., Silva P., Uscategui R.A.R., Padilha-Nakaghi L.C., Lima B.B., Miranda B.S.P.d., Feliciano M.A.R. (2023). Ultrasonography and elastography of the brain and cerebellum of English Bulldog fetuses. Theriogenology.

[67] Quarello E., Lacoste R., Mancini J., Melot-Dusseau S., Gorincour G. (2015). Feasibility and reproducibility of ShearWave(TM) elastography of fetal baboon organs. Prenat. Diagn..

[68] Hwang M., Zhang Z., Katz J., Freeman C., Kilbaugh T. (2022). Brain contrast-enhanced ultrasonography and elastography in infants. Ultrasonography.

[69] Kolarovszki B., Zubor P., Kolarovszka H., Benco M., Richterova R., Matasova K. (2013). The assessment of intracranial dynamics by transcranial Doppler sonography in perioperative period in paediatric hydrocephalus. Arch. Gynecol. Obstet..

[70] Hanlo P.W., Gooskens R.H.J.M., Nijhuis I.J.M., Faber J.A.J., Peters R.J.A., van Huffelen A.C., Tulleken C.A.F., Willemse J. (1995). Value of transcranial Doppler indices in predicting raised ICP in infantile hydrocephalus. Child’s Nerv. Syst..

[71] Barletta A., Balbi M., Surace A., Caroli A., Radaelli S., Musto F., Saruggia M., Mangili G., Gerevini S., Sironi S. (2021). Cerebral superb microvascular imaging in preterm neonates: In vivo evaluation of thalamic, striatal, and extrastriatal angioarchitecture. Neuroradiology.

[72] Freeman C.W., Hwang M. (2022). Advanced ultrasound techniques for neuroimaging in pediatric critical care: A review. Children.

[73] Couture O., Hingot V., Heiles B., Muleki-Seya P., Tanter M. (2018). Ultrasound Localization Microscopy and Super-Resolution: A State of the Art. IEEE Trans. Ultrason. Ferroelectr. Freq. Control.

[74] Viessmann O.M., Eckersley R.J., Christensen-Jeffries K., Tang M.X., Dunsby C. (2013). Acoustic super-resolution with ultrasound and microbubbles. Phys. Med. Biol..

[75] Ackermann D., Schmitz G. (2016). Detection and Tracking of Multiple Microbubbles in Ultrasound B-Mode Images. IEEE Trans. Ultrason. Ferroelectr. Freq. Control.

[76] Poelma C. (2017). Ultrasound Imaging Velocimetry: A review. Exp. Fluids.

[77] Kim H.B., Hertzberg J.R., Shandas R. (2004). Development and validation of echo PIV. Exp. Fluids.

[78] Zhang W., Yi H., Cai B., He Y., Huang S., Zhang Y. (2022). Feasibility of contrast-enhanced ultrasonography (CEUS) in evaluating renal microvascular perfusion in pediatric patients. BMC Med. Imaging.

[79] Lee M.S., Lee J.S., Kim B.S., Kim D.R., Kang K.S. (2021). Quantitative Analysis of Pancreatic Fat in Children with Obesity Using Magnetic Resonance Imaging and Ultrasonography. Pediatr. Gastroenterol. Hepatol. Nutr..

